# Asialoerythropoietin ameliorates bleomycin-induced acute lung injury in rabbits by reducing inflammation

**DOI:** 10.3892/etm.2014.1960

**Published:** 2014-09-12

**Authors:** AKINAGA SONODA, NORIHISA NITTA, KEIKO TSUCHIYA, HIDEJI OTANI, SHOBU WATANABE, KENICHI MUKAISHO, YUKI TOMOZAWA, YUKIHIRO NAGATANI, SHINICHI OHTA, MASASHI TAKAHASHI, KIYOSHI MURATA

**Affiliations:** 1 Department of Radiology, Shiga University of Medical Science, Otsu, Shiga 520-2192, Japan; 2 Department of Pathology, Shiga University of Medical Science, Otsu, Shiga 520-2192, Japan

**Keywords:** asialoerythropoietin, acute lung injury, rabbit, bleomycin, score

## Abstract

Acute lung injury, a critical illness characterized by acute respiratory failure with bilateral pulmonary infiltrates, remains unresponsive to current treatments. The condition involves injury to the alveolar capillary barrier, neutrophil accumulation and the induction of proinflammatory cytokines followed by lung fibrosis. In the present study, a rabbit model of bleomycin-induced acute lung injury was established to examine the effects of asialoerythropoietin (AEP), an agent with tissue-protective activities, on pulmonary inflammation. Six Japanese white rabbits were randomly divided into two equal groups. Acute lung injury was induced in all rabbits by intratracheally injecting bleomycin. The control group was injected with bleomycin only; the experimental (AEP) group was injected intravenously with AEP (80 μg/kg) prior to the bleomycin injection. Computed tomography (CT) studies were performed seven days later. The CT inflammatory scores of areas exhibiting abnormal density and the pathological inflammatory scores were recorded as a ratio on a 7×7 mm grid. The CT and pathological inflammatory scores were significantly different between the control and AEP groups [122±10 and 16.3±1.5 (controls) vs. 71±8.5 and 9.7±1.4 (AEP), respectively; P<0.01]. Thus, the present study revealed that AEP prevents bleomycin-induced acute lung injury in rabbits.

## Introduction

Acute lung injury and acute respiratory distress syndrome (ARDS) are life-threatening conditions involving acute respiratory failure associated with extensive pulmonary infiltrates (1–3). The infiltration of neutrophils and activation of proinflammatory cytokines leads to the destruction of the alveolar capillary barrier and subsequent lung fibrosis. Due to the persistent respiratory failure, the morbidity and mortality rates of affected patients remain high (1,3). The reported overall mortality rate for individuals with acute lung injury is ~30% (3), and the currently available treatment for patients with acute lung injury and ARDS is primarily palliative (2,4).

Erythropoietin (EPO) is the major regulator of erythroid precursor cells. It elicits cytoprotective responses and has been used in experiments to treat organ injury and angiogenesis, inhibit apoptosis and fibrosis, and enhance tissue regeneration (5–10). According to Kakavas *et al* (11), advances in the understanding of the biological and biochemical activities of EPO may be useful in the management of patients with acute lung injury and ARDS.

Yokomaku *et al* (12) revealed that the engineered EPO derivative asialoerythropoietin (AEP), whose half-life is extremely short, is non-hematopoietic but appears to retain extra-hematopoietic effects and may, similarly to native EPO, be of potential use in the treatment of patients with acute lung injury and ARDS. In the present study a rabbit model of bleomycin-induced acute lung injury was established to determine whether AEP ameliorated the effects of pulmonary inflammation.

## Materials and methods

### Experimental procedures

The procedures in the present study were approved by the Animal Experimentation Committee and performed according to the Animal Care Guidelines of the Shiga University of Medical Science (Otsu, Japan). The rabbits were anesthetized with intramuscular injections of a mixture of ketamine hydrochloride (25 mg/kg Ketalar^®^ 50; Sankyo Yell Yakuhin Co., Ltd., Tokyo, Japan) and medetomidine hydrochloride (0.1 mg/kg Domitor^®^, Meiji Seika Co., Ltd., Tokyo, Japan) prior to all experimental procedures. The rabbits were housed in a temperature-controlled room (24±1°C) under a 12-h light/dark cycle. Standard laboratory chow was available *ad libitum*.

### Administration of bleomycin and AEP

Six adult female Japanese white rabbits (3.0 kg; Japan SLC Inc., Tokyo, Japan) were randomly divided into two equal groups (n=3). One group served as the control and was treated with bleomycin only. At 30 min prior to the bleomycin injection, the second group (AEP group) was pretreated with 80 ng/g AEP (Chugai Pharmaceutical Co., Ltd., Tokyo, Japan) delivered by an intravenous bolus injection via the auricular vein.

All rabbits were anesthetized with ketamine hydrochloride and medetomidine hydrochloride. Bleomycin hydrochloride (30 mg; Sigma-Aldrich, Tokyo, Japan) was dissolved in 2 ml physiological saline and 8 U/kg bleomycin was subsequently injected into the trachea using a 22-gauge indwelling needle. To obtain equal drug distribution throughout the lungs, four injections of 2 U/kg each were delivered to each rabbit in the prone and dorsal positions, on the right and left lateral sites.

### White blood cell (WBC) measurements

A total of 5 ml blood was collected from the auricular vein of each rabbit. WBC counts were obtained prior to any treatment and seven days post-treatment using a Celltac-α^™^ analyzer (MEK-6358; Nihon Kohden, Tokyo, Japan).

### Computed tomography (CT) studies

Images were captured on a four-row multidetector CT scanner (Toshiba Medical System Corporation, Otawara, Japan) prior to, immediately after and seven days after the administration of bleomycin. The scanning parameters were as follows: X-ray tube voltage, 120 kV; X-ray tube current, 50 mA; collimation, 1 mm; field of view, 100 mm; and helical pitch, 0.8. The CT images were subsequently reconstructed; horizontal 1-mm cross-sections were constructed at 5-mm intervals from the apex to the bottom of the lungs in the lung field.

### Scoring of CT images

**Figure 1:**
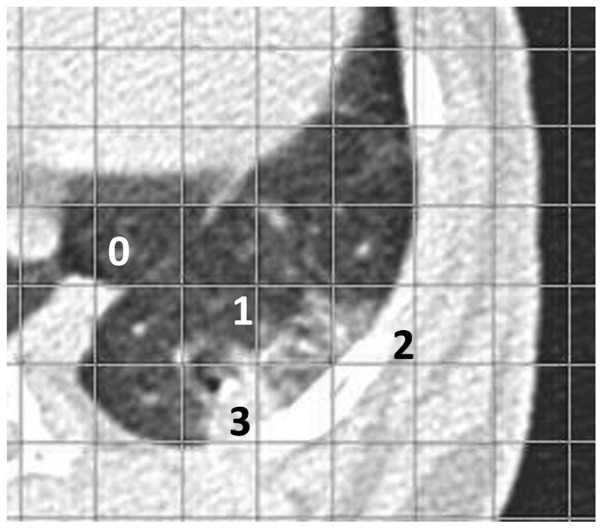
Areas with abnormal density on the computed tomography images. Consolidation, homogeneous ground-glass opacity and reticulolinear shadows were scored as a ratio of the grid as follows: 0, normal; 1, abnormal area <1/4; 2, abnormal area ≥1/4 but <1/2; and 3, abnormal area ≥1/2.

Two of the authors analyzed the images with Microsoft PowerPoint 2010 (Microsoft Corporation, Redmond, WA, USA) using a 7×7 mm grid. Areas with abnormal density on the CT images, reflective of consolidation, homogeneous ground-glass opacity and reticulolinear shadows, were scored as a ratio of the grid as follows: 0, normal; 1, abnormal area <1/4; 2, abnormal area ≥1/4 but <1/2; and 3, abnormal area ≥1/2 (Fig. 1).

### Histopathological examination

Rabbits were sacrificed via an injection of pentobarbital (Sumitomo Dainippon Pharma Co. Ltd., Tokyo, Japan) into the heart on day 7 post-treatment. The lungs were resected, fixed in formaldehyde and cut into 4-μm slices using a LEICA SM2000 R sliding microtome (Leica Microsystems, Tokyo, Japan). Consecutive slices were mounted on glass slides and stained with hematoxylin and eosin. One cross-section on each glass slide was selected from the center of the craniocaudal axis in the bilateral anterior and posterior lobes.

### Scoring of pathological specimens

**Figure 2:**
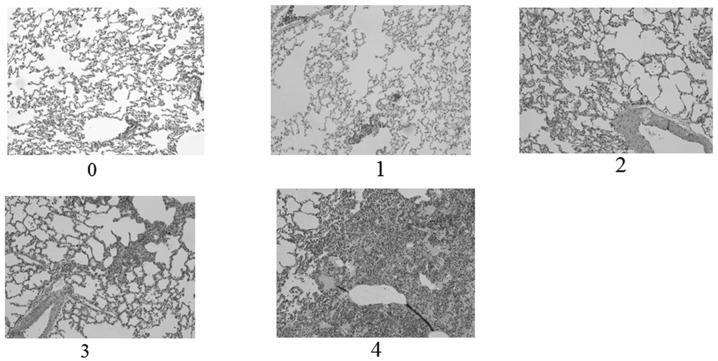
Scoring of pathological specimens. Non-overlapping fields from each lung specimen were evaluated for inflammation as follows: 0, no inflammation; 1, focal interstitial infiltrates; 2, diffuse interstitial infiltrates; 3, focal alveolar infiltrates; and 4, confluent alveolar infiltrates. Microscopic images (hematoxylin and eosin staining; magnification, ×100).

Using a Nikon ECLIPSE 90i (Nikon, Otawara, Japan), images (magnification ×100) were evaluated by two blinded readers who consensually scored the degree of inflammatory cell infiltration in the alveolar wall and alveoli. A total of 10 sequential, non-overlapping fields from each lung specimen were evaluated for inflammation as follows: 0, no inflammation; 1, focal interstitial infiltrates; 2, diffuse interstitial infiltrates; 3, focal alveolar infiltrates; and 4, confluent alveolar infiltrates (Fig. 2).

### Statistical analysis

SPSS 20.0 software (IBM Corp., Tokyo, Japan) was used for data analysis. CT and pathology results of the two rabbit groups were compared using the Student’s t-test. P<0.05 was considered to indicate a statistically significant difference.

## Results

### WBC count

**Figure 3:**
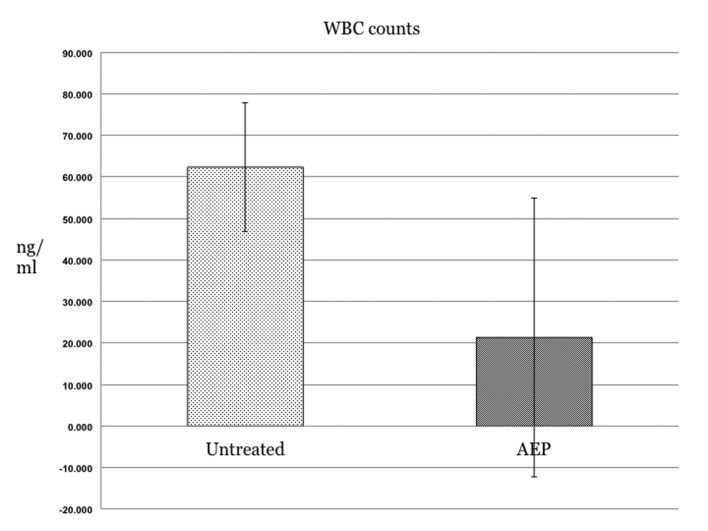
WBC counts in the AEP-treated and -untreated rabbits. The WBC counts were lower in the AEP-treated rabbits than those in the control rabbits (21.3±33.6 vs. 62.3±15.5 ng/ml, respectively; P=0.127). WBC, white blood cell; AEP, asialoerythropoietin.

The WBC count was lower in the AEP group than that in the control group (21.3±33.6 vs. 62.3±15.5 ng/ml, respectively). At P=0.127, the difference was not statistically significant (Fig. 3).

### Inflammatory scores based on CT images

**Figure 4:**
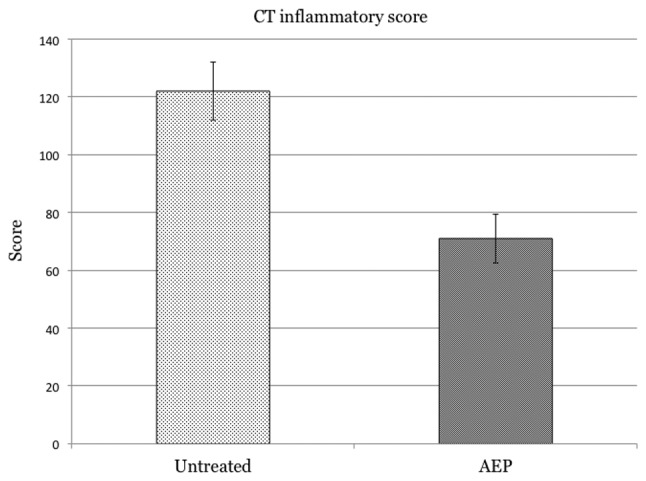
Inflammatory scores in the AEP-treated and -untreated rabbits. The inflammatory score was significantly higher in the control rabbits than that in the AEP-treated rabbits (122±10 vs. 71±8.5, respectively; P=0.003). AEP, asialoerythropoietin.

The inflammation score was lower in the AEP-treated group than that in the control group (71±8.5 vs. 122±10, respectively; Fig. 4). The difference between the two groups was significant (P=0.003).

### Pathological inflammatory scores

**Figure 5:**
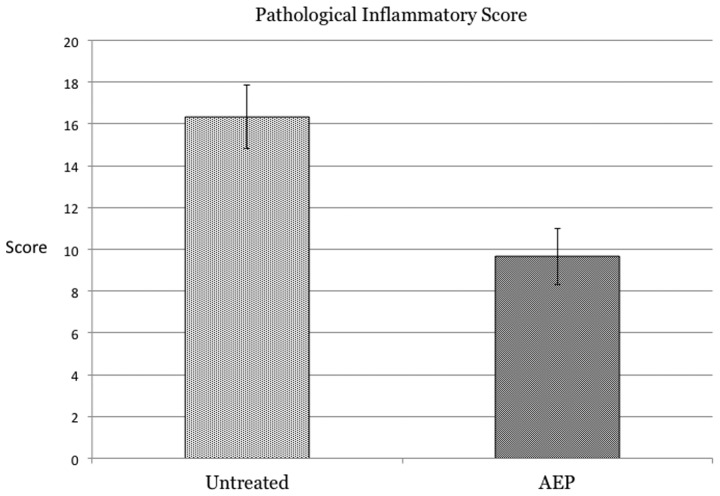
Pathological inflammatory scores. The inflammatory scores were higher in the control rabbits than those in the AEP-treated rabbits (16.3±1.5 vs. 9.7±1.4, respectively; P=0.005). AEP, asialoerythropoietin.

Macroscopically, the two groups did not differ in inflammatory score. However, based on the microscopic results, the average inflammatory score of the control rabbits was higher than that of the AEP-treated rabbits (16.3±1.5 vs. 9.7±1.4; P=0.005; Fig. 5).

## Discussion

The present study demonstrated that AEP inhibited the induction of inflammatory cells in the interstitial and alveolar tissue of rabbits subjected to bleomycin-induced acute lung injury. The direct injection of bleomycin into the airway caused injury to the lung epithelium and endothelium and elicited an inflammatory response (13).

Acute lung injury and ARDS have an early and late phase. The early phase is characterized by an inflammatory response and the late, fibroproliferative phase is characterized by collagen deposition with tissue remodeling (14). The contribution of inflammatory cells to acute lung injury has been previously demonstrated (15). Activated neutrophils release various cytotoxic mediators, including reactive oxygen species.

In the present study, no significant difference was observed between the WBC counts of the control and AEP-treated rabbits. Even prior to the administration of bleomycin, two of the six rabbits manifested high WBC counts (108 and 128 ng/ml, respectively); however, CT studies confirmed that none had a pre-existing inflammatory lung disease. As the rabbits were of a mature age, the potential presence of inflammation in extra-pulmonary organs cannot be ignored.

CT and microscopic studies revealed a significant difference in the inflammatory scores of the control and AEP-treated rabbits. On the CT images, the scores assigned to the areas of abnormal density, reflective of severe alveolar and interstitial edema and neutrophil infiltration, were significantly lower for the AEP-treated rabbits than those for the control animals. Microscopic inspection also revealed that the degree of infiltration by inflammatory cells was lower in the AEP-treated rabbits than that in the control group.

The results of the current study suggested that AEP exerted anti-inflammatory effects on the rabbit model of bleomycin-induced lung injury. Shang *et al* (8) reported that the pretreatment of rats with EPO inhibited the production of tumor necrosis factor-α and interleukin-1β, thereby decreasing the degree of pulmonary edema and infiltration by neutrophils in the lung tissue. The mechanism(s) of action of EPO and AEP may be similar. AEP may exert ameliorative effects in the early phase of acute lung injury and ARDS and may inhibit collagen deposition and tissue remodeling in the late phase.

In the present study the ameliorative effects of AEP were less pronounced than expected. AEP was administered 30 min prior to the injection of 80 ng/g bleomycin following protocols previously published (12,16), and the effect of this particular time of delivery and dose of AEP requires further study.

To the best of our knowledge, no studies have been published on the metabolism of AEP. Although Imai and Osawa (17) observed that AEP binds to its receptors faster than native EPO, the delivery of AEP at 30 min prior to the administration of bleomycin may not have allowed sufficient time for the manifestation of its ameliorative effects.

The present preliminary study revealed that pretreatment with AEP attenuated pulmonary inflammation in a rabbit model of bleomycin-induced acute lung injury. Further studies are underway to determine the role of the timing of delivery and dose of AEP on its effects and to examine whether AEP may be a potential therapeutic agent to treat acute lung injury. In practice, since the mortality rate is high in elderly patients with ARDS, an AEP-based treatment may be effective at improving the survival rate of patients.
